# Evaluation of anti-fatigue property of the extruded product of cereal grains mixed with *Cordyceps militaris* on mice

**DOI:** 10.1186/s12970-017-0171-1

**Published:** 2017-06-02

**Authors:** Lei Zhong, Liyan Zhao, Fangmei Yang, Wenjian Yang, Yong Sun, Qiuhui Hu

**Affiliations:** 1 College of Food Science and Technology, Nanjing Agricultural University, Nanjing, 210095 China; 2 College of Food Science and Engineering, Nanjing University of Finance and Economics, Nanjing, 210023 China; 3 Beijing Academy of Food Sciences, Beijing, 100068 China

**Keywords:** Anti-fatigue, Extruded product, Cereal grains, *Cordyceps militaris*

## Abstract

**Background:**

Fatigue is a biological phenomenon that involves a feeling of extreme physical or mental tiredness that could potentially cause some severe chronic diseases. Recently, diet therapy has provided a new alternative to alleviate physical fatigue. In our previous study, addition of *Cordyceps militaris* (*C. militaris*) into an extruded product was shown to provide high nutrition and unique flavors; however, little is known whether this product has some scientific evidence regarding anti-fatigue property. The purpose of this study was to evaluate the anti-fatigue effects of extruded products of cereal grains (EC) and EC mixed with *C. militaris* (ECC).

**Methods:**

The mice were divided into seven groups: one group received distilled water (Control group, *n* = 20), and the other groups received different dosages of EC (5, 10 and 20 g/kg body weight, *n* = 20 per group) or of ECC (5, 10 and 20 g/kg body weight, *n* = 20 per group) solution in water. All of the mice were administered with distilled water, EC or ECC continuously for 30 days by gavage and the anti-fatigue activity was evaluated using a weight-loaded swimming test, along with assessments of fatigue-related indicators. The mode of fighting fatigue was investigated by determining changes in exercise endurance and biochemical markers, including exhaustive swimming time, lactate dehydrogenase (LDH), blood lactic acid (BLA), creatine kinase (CK), blood urea nitrogen (BUN), malondialdehyde (MDA), glutathione peroxidase (GSH-Px), superoxide dismutase (SOD), catalase (CAT), and hepatic and muscle glycogen levels.

**Results:**

EC and ECC prolonged the swimming endurance time of mice compared to the control. The content of BLA at high dose of ECC group (20 g/kg) was significantly lower than that in the negative control group. CK, BUN and MDA levels were significantly reduced by treatment with EC and ECC compared to the negative control, while the low and middle dose of EC had no significant effect on MDA levels. Additionally, only the middle and high dose of EC (10, 20 g/kg) could significantly decrease the BUN level. EC and ECC treatments increased glycogen, LDH, SOD, CAT and GSH-Px levels. Low and middle dose of EC had no significant effects on muscle glycogen. Moreover, low dose of EC could increase the level of SOD but it was not statistically significant. Compared to the EC treatment groups, ECC demonstrated the efficacy of anti-fatigue potential, particularly at a high dose of ECC, the best performance in relieving fatigue.

**Conclusions:**

These results suggest that EC and ECC could prevent exercise-induced fatigue in mice and ECC provided a better effect. In addition, *C. militaris* in ECC might play a crucial role in the anti-fatigue activity of ECC.

## Background

Physical fatigue is commonly defined as an inability to sustain or maintain voluntary activities, which is considered to be associated with the decline of physiology [1, 2]. Regular exercise, a balanced diet, and complementary and alternative medicine (CAM) are reported to effectively relieve fatigue, including providing energy substrates and enhancing elimination of metabolites. Among these approaches, CAM dietary therapy has been widely used in medical treatment and health care due to its minor side effects [3, 4].

Whole cereal grains as healthy foods have gained popularity due to their underlying protection against diet-related disorders, such as fatigue, obesity, diabetes and even cancers [5–8]. In addition, the limited amino acids of cereal and legume were lysine and methionine respectively, while mixed cereal grains are able to compensate for deficiencies in the content and constitution of essential amino acids from a single source. Currently, mixed cereal grains have been applied to design and produce nutritional foods that are rich in dietary fiber and protein [9].

In recent years, research on extruded products including edible mushrooms has received increased attention in order to obtain an improved nutritional value, texture, flavor and digestion of food under conditions of thermal force and ultra-high pressure [10–13]. *C. militaris* is a fungus renowned for its health-promoting properties, as well as for its medicinal use in Southeast Asia [14]. In a previous study, *C. militaris* was demonstrated to be a high quality protein and rich amino acid, which contains 29.43% protein, 2.51% fat, 1.20% dietary fiber, 37.52% carbohydrate and 0.051% cordycepin etc. Moreover, its bioactive components such as cordycepin, polysaccharides, ergosterol, and adenosine, play a key role in health care, and are used for various medicinal purposes such as anti-aging, anti-tumor, anti-cancer and anti-leukemic etc. [15–17]. *C. militaris* was also often used in the formulation of functional foods. Inclusion of *C. militaris* into chicken essence products could be nutritional and healthy for consumers [18]. Recently, the aqueous extract of *C. militaris* had been demonstrated to possess the significant effect on resisting oxidation and relieving fatigue in mouse model [19]. *Cordyceps sinensis* (*C. sinensis*), widely accepted as tonic and healthy foods in Asia, has received more interests due to its biological activity. Many studies reported that *C. sinensis* has the prominent impacts on analgesic, antitumor, free radical scavenging, and immune modulation [20]. Compared with *C. sinensis*, the contents of cordycepin, adenine, guanosine and uracil were even higher in *C.militaris*, while cordycepic acid, amino acid, polysaccharides and inorganic elements levels were lower. Natural *C. sinensis* is expensive and rare in nature. *C. militaris* has medicinal properties similar to those of *C. sinensis* and could potentially be the alternatives for *C. sinensis* in health supplements. Due to high cost in the development of pharmaceutical drugs, more and more research interests have been put on natural products, which are proved to be safer and more efficacious with less side effects on body. Thus, *C. militaris* could be used as a high-quality raw material of functional food [21].

Weight-loaded swimming test is the most widely used method to evaluate anti-fatigue properties of compounds through assessing the reduction of muscular force or the exhaustion of contractile function [22]. On the basis of the test, the resistant fatigue mechanisms of compounds could be investigated through a series of biochemical parameters. Several theories have been proposed to explain sports fatigue, including the “exhaustion theory [23]”, “radical theory [24]”, “clogging theory”, “homeo-stasis disturbance theory”, “mutation theory” and “protective inhibition theory [25]”. Among these theories, the “exhaustion theory”, “clogging theory” and “radical theory” have drawn particular attention, which could explain the anti-fatigue effect of the extruded products by evaluating the values of biochemical criteria. The “exhaustion theory” proposed that depletion of muscle and liver glycogen could lead to the ocurrence of fatigue. However, “clogging theory”, the excessive accumulation of BLA and BUN would cause metabolic disorders, which eventually result in fatigue. “Radical theory” shows that intense exercise could produce an imbalance between the body’s oxidation system and its anti-oxidation system. Muscle cells have the defense mechanisms to scavenge reactive oxygen species, such as SOD, GSH-Px and CAT, and to protect cells against exercise-induced oxidative injury. Currently, research on healthy foods, such as *C. militaris, Panax quinquefolium and fermented rice bran*, have achieved great progress in relieving fatigue by reducing the CK and BLA contents and increasing glycogen and GSH-Px concentrations [4, 26, 27]. In our previous study, the proportions of cereal grains in a mixed powder were optimized by linear programming. Moreover, ECC was previously shown to possess profound characteristic features, such as the improvement in texture, nutrition and flavors. However, the physiological effects or health functions of ECC remain unclear.

In the previous study, *C. militaris* and blended powders from five cereal grains were prepared; a twin screw extruder was used to produce the extruded products. We hypothesized that EC and ECC could alleviate fatigue in mice and adding *C. militaris* could enhance the anti-fatigue effect of EC. To test the hypothesis, we established the mouse model of sports fatigue and studied the changes in anti-fatigue and anti-oxidant activities of EC and ECC. The swimming endurance time was determined as an indicator of athletic capability in mice, which could intuitively reflect the ability of resistance to fatigue. Following swimming test, mice were subject to a series of biochemical analysis in order to further explore the anti-fatigue mechanisms of EC and ECC such as measuring LDH and CK as well as the levels of BUN, BLA, MDA, muscle and liver glycogen. In addition, the anti-oxidant enzymes including SOD, CAT and GSH-Px were also measured.

## Methods

### Plant materials and reagents

Rice, adlay, red beans, glutinous rice and soybeans were purchased from a local supermarket. *C. militaris* was provided from Yan Cheng, Jiangsu Province, China.

A liver/muscle glycogen assay kit, urea assay kit, lactate dehydrogenase assay kit, GSH-Px assay kit, MDA assay kit, lactic acid assay kit, SOD assay kit, CAT assay kit and CK assay kit were obtained from Nanjing Jiancheng Bioengineering Institute, Jiangsu Province, China.

### Preparation of extruded products

In our previous study, the composition of the blended cereal grains was calculated using linear programming in Microsoft Excel: 60% rice, 5% adlay, 10% red beans, 10% glutinous rice and 15% soybeans. The grains were mixed with *C. militaris* at a ratio of 10:1 (grains:*C. militaris*) on a dry-to-dry weight basis. The moisture content of the mixed samples was adjusted to 16% by spraying the samples with calculated amounts of water, and the samples were then stored at 4 °C overnight. The extrusion temperature (80 °C−90 °C−120 °C−140 °C−165 °C), screw speed (180 rpm) and feed rate (15 rpm) were set by twin screw extrusion (DSE-29/40D, Brabender Co. Ltd., Germany). Wet materials were processed in the barrel into extruded products, and packed under vacuum.

### Animals and experimental design

Approval for the experimental protocol from institutional animal ethical committee was obtained before initiation of the study from Nanjing Agricultural. The study was conducted in the laboratory of the animal center of Nanjing Agricultural University (Nanjing, China) and all of the mice were euthanized at the end of experiment. C57BL/6 male mice weighing approximately 18–22 g (seven weeks old, specific pathogen-free grade, SPF) were housed under controlled conditions, including the room temperature (25 °C), relative humidity (50%), and air flow conditions, with a fixed 12-h light–dark cycle during the experiment. All the mice were allowed free access to water and a standard diet.

The mice were randomly divided into seven groups, with 20 mice in each group. Mice administered with distilled water (20 g/kg body weight) by gavage served as the negative control group (NCG). The treatment mice were fed with EC or ECC by a feeding atraumatic needle once per day for 30 days at a low-dose 5 g/kg (EC-L and ECC-L), medium dose of 10 g/kg (EC-M and ECC-M) and a high dose of 20 g/kg (EC-H and ECC-H). All the mice were fed normal laboratory food and they drank water freely. The high dose was calculated at 20 g/kg body weight based on 150 g cereal grains intake by the adult (60 kg of body weight), and the low- and middle-dose values were 5 g/kg and 10 g/kg, respectively. All the extruded products were prepared by dissolving in the same volume of distilled water.

### Weight-loaded swimming test

The weight-loaded swimming test was carried out with some modifications [28–30]. Following the last treatment with EC, ECC or distilled water, ten mice in each group rested for 30 min and were placed in the swimming pool (100 cm × 50 cm × 40 cm, 25 ± 1.5 °C), where the mice were attached to a brass ring of 5% bodyweight and could only support themselves by touching the bottom with their feet. Exhaustion was determined by the time on observing their loss of coordinated movements and failure to swim. Endurance time was recorded by the experimenters until the mice were completely exhausted and failed to return to surface to breathe within 10s. The exhausted mice were removed from the pool, dried with gauze and returned to their cages.

### Sample collection

**Fig. 1:**
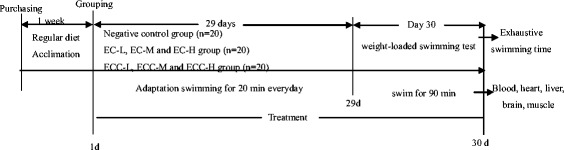
Experiment scheme

The rest of the ten mice were taken out of each group to swim for the determination of organ index and analyses of glycogen and blood biochemical parameters. After the final treatment, they were forced to swim to exhaustion without loads for 90 min in the same pool and then rested for an hour. The mice subjected to forced swimming test without loads for 90 min in the same pool and then rested for an hour. These mice were anesthetized with pentobarbital sodium to collect blood, organ and tissue. Blood samples were collected through their eyeballs into heparinized tubes and tubes without anticoagulant. Serum samples were prepared by centrifugation at 1000 × g, 4 °C for 15 min and were then tested to determine the concentrations of LDH, CK, BUN, MDA, GSH-Px, SOD and CAT. Blood plasma was prepared by centrifugation at 1000 × g, 4 °C for 10 min, and then tested to measure the BLA content. Blood analysis was tested in accordance with the instructions for each kit. Liver, brain and leg muscles were quickly dissected. All the organs and tissues were washed in ice-saline and then dried with filter paper for the determination of the organ index. Livers and leg muscles were tested to evaluate the changes in glycogen levels. All the samples were frozen in liquid nitrogen and kept at−80 °C until the determination was performed. The experiment scheme was shown in Fig. 1.

### Determination of the organ index

Livers, brains and hearts were weighed using electronic scales. The formula of the organ index is shown below:

$$ I=\frac{100\ast g}{G} $$

where *I* is the organ index, *g* is the weight of the organ, and *G* is the weight of the mouse.

### Statistical analysis

Means ± SD (*n* = 10) are shown in Tables 1 and 2, and the error bars are marked in the figures. One-way analysis of variance (ANOVA) was used to detect statistical significance followed by post hoc multiple comparisons using SPSS 22.0 software (IBM corporation, USA). *P < 0.05* was considered statistically significant.

**Table 1:** Effects of EC and ECC on body weight changes and organ index in mice

Group	Body weight (g)				Organ index		
	Initial	Seventh day	Intermediate	Final	Liver	Brain	Heart
NCG	18.894 ± 0.845	19.604 ± 0.462	21.592 ± 0.504	23.097 ± 0.592	0.030 ± 0.020	0.055 ± 0.066	0.009 ± 0.005
EC-L	19.159 ± 0.445	20.436 ± 0.773	22.077 ± 0.913	23.082 ± 1.128	0.023 ± 0.008	0.047 ± 0.066	0.015 ± 0.038
EC-M	19.274 ± 1.327	20.135 ± 1.304	21.954 ± 1.166	23.392 ± 0.960	0.022 ± 0.007	0.050 ± 0.024	0.013 ± 0.041
EC-H	19.725 ± 0.736	20.783 ± 1.141	22.583 ± 1.386	23.537 ± 1.568	0.021 ± 0.018	0.046 ± 0.002	0.012 ± 0.008
ECC-L	19.774 ± 0.793	20.647 ± 0.898	22.581 ± 0.956	23.223 ± 1.081	0.025 ± 0.011	0.048 ± 0.082	0.016 ± 0.079
ECC-M	19.462 ± 0.833	20.220 ± 0.864	22.149 ± 0.948	22.993 ± 1.364	0.024 ± 0.010	0.049 ± 0.019	0.011 ± 0.115
ECC-H	19.528 ± 1.087	20.687 ± 1.118	22.356 ± 1.525	23.468 ± 1.504	0.027 ± 0.005	0.051 ± 0.060	0.014 ± 0.098

Treatment with distilled water (20 g/kg), EC and ECC (5 g/kg, 10 g/kg, 20 g/kg) for 30 days; the body weight was recorded before experiment (initial), the seventh day, after 14 days (intermediate) and after 28 days (final); after exhaustive swimming, the liver, brain and heart index were detected. Values are means ± standard deviations

**Table 2:** Effects of EC and ECC on glycogen levels from muscle and liver

Group	Muscle glycogen (mg/g muscle)	Liver glycogen (mg/g liver)
NCG	0.093 ± 0.009	0.081 ± 0.003
EC-L	0.207 ± 0.078	1.062 ± 0.083*
EC-M	0.213 ± 0.103	1.244 ± 0.090*
EC-H	0.274 ± 0.051*	1.326 ± 0.079*
ECC-L	0.425 ± 0.007*^a^	2.078 ± 0.055*^a^
ECC-M	0.476 ± 0.010*^b^	3.534 ± 0.083*^b^
ECC-H	0.533 ± 0.021*^c^	4.312 ± 0.131*^c^

Treatment with distilled water (20 g/kg), EC and ECC (5 g/kg, 10 g/kg, 20 g/kg) for 30 days; After exhaustive swimming, the muscle and liver glycogen level were determined. Values are means ± standard deviations (*n* = 10). (*) *p* < 0.05 indicate statistically significant differences versus NCG; (a), (b), (c) *p* < 0.05 indicate statistically significant differences versus EC-L, EC-M and EC-H group respectively

## Results

### Effects of EC and ECC on body weights and on the organ index

Mice weight were recorded following each treatment during the experiment (Table 1). No significant difference were observed in the initial body weights among the groups (*P > 0.05*). However, mice in the treatment groups appear to be higher than those in the NCG, but the difference was not significant (*P > 0.05*). In addition, there were no significant changes in the organ index for all the mice among the groups (*P > 0.05*).

### Effects of EC and ECC on exhaustive swimming capacity

**Fig. 2:**
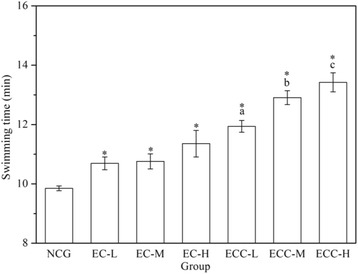
Effects of EC and ECC on exhaustive swimming time in mice. Male C57BL/6 mice were divided into 7 groups that were given distilled water or different dosages of EC or ECC (5 g/kg, 10 g/kg or 20 g/kg), and the exhaustive swimming test was performed. Values are expressed as the mean ± SEM. The error bars represent one standard deviation, and (*) *p* < 0.05 indicate statistically significant differences versus NCG; (*a*), (*b*), (*c*) *p* < 0.05 indicate statistically significant differences versus EC-L, EC-M and EC-H group respectively (the same below)

The exhaustive swimming capacities are shown in Fig. 2. For EC- and ECC-supplemented groups, the exhaustive swimming time was longer than that for the NCG (*P < 0.05*) and increased with a rising concentrations of EC and ECC. In addition, compared to the EC-H group, the exhaustive swimming time in the ECC-H group (20 g/kg body weight) was prolonged by 18.21%, which showed that ECC-H mice had the most significant effect on increasing exhaustive swimming ability.

### Effects of EC and ECC on glycogen levels

Table 2 shows that glycogen levels (muscle and liver glycogen) in the EC- and ECC-supplemented groups were significantly higher than those in the NCG (*P < 0.05*). At the same dose, the mice receiving ECC had significantly higher glycogen levels than those in the EC-supplemented groups (*P < 0.05*). Compared to EC-H group, the muscle glycogen levels in the ECC-L, ECC-M and ECC-H groups were increased by 55.11%, 73.72%, and 94.53%, and the liver glycogen levels also had an increase of 56.71%, 166.52% and 225.19%, respectively.

### Effects of EC and ECC on the serum LDH and CK activities

**Fig. 3:**
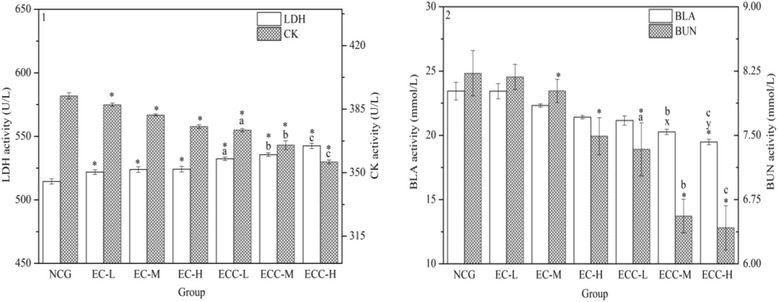
Effects of EC and ECC on LDH and CK activities (1) and on BLA and BUN levels (2) in mice. Male C57BL/6 mice were treated with EC or ECC (5 g/kg, 10 g/kg or 20 g/kg), and LDH and CK activities (1) and BLA and BUN levels (2) were measured. Values are expressed as the mean ± SEM. The error bars represent one standard deviation, and (x), (y) *p* < 0.05 indicate statistically significant differences versus EC-supplemented groups (the same below)

As shown in Figs. 1, 2, and 3, following swimming test, LDH activity in the ECC-supplemented groups increased significantly compared to that in the NCG. Under the same dosage, the LDH levels in the ECC-supplemented groups were higher than those in the EC-supplemented groups (*P < 0.05*). Compared to EC-H group, LDH contents of mice in the ECC-L, ECC-M and ECC-H groups increased by 1.57%, 2.18% and 3.50%, respectively. In addition, there was no significant dose-dependent fatigue relief in the EC-supplemented groups.

CK activity was determined in order to evaluate the effects of EC and ECC on anti-fatigue capacity (Figs. 1, 2, and 3). Compared with the NCG, significant decreases (*P < 0.05*) in the CK level were observed in the EC- and ECC-supplemented groups. Under the same dosage, CK activities in the ECC-supplemented groups were significantly (*P < 0.05*) lower than those in the EC-supplemented groups. Compared with the EC-H group, the CK concentration in mice in the ECC-L, ECC-M and ECC-H group decreased by 0.51%, 2.72% and 5.18% respectively, indicating that ECC-H resulted in the most significant reduction in the CK level.

### Effects of EC and ECC on BLA and BUN levels and on MDA activity

Compared with the NCG, the BLA concentration in the ECC-H treatment groups showed a significant reduction, as shown in Figs. 2 and 3. The mice in the ECC-M and ECC-H groups had lower (*P < 0.05*) BLA levels than those in the EC-supplemented groups. Compared with the EC-H group, the BLA levels in the ECC-L, ECC-M and ECC-H groups evidently decreased (*P < 0.05*) by 1.23%, 5.38% and 9.06%, respectively.

BUN is a sensitive indicator of exercise capacity (Figs. 2 and 3). Compared to the NCG, the BUN levels in the EC-M, EC-H group and ECC-supplemented groups were significantly (*P < 0.05*) reduced. The BUN levels in the ECC-supplemented groups were lower than those in the EC-supplemented groups. Additionally, compared to the EC-H group, the BUN level in the ECC-L, ECC-M and ECC-H group significantly decreased by 2.05%, 12.45% and 14.29%, respectively.

**Fig. 4:**
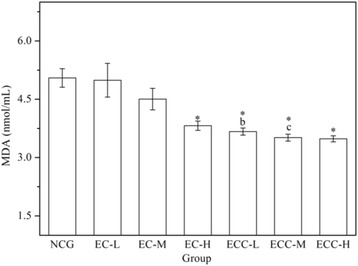
Effects of EC and ECC on MDA activity in mice after exhaustive swimming. Values are expressed as the mean ± SEM. The error bars represent one standard deviation

As shown in Fig. 4, the serum MDA levels in the EC-H and ECC treatment groups were significantly lower (*P < 0.05*) than those in the NCG. Compared with the EC-H group, the serum MDA levels of the mice in the ECC-L, ECC-M and ECC-H groups decreased by 3.90%, 8.05% and 8.83%, respectively. Additionally, the data showed that EC-H and ECC treatment groups showed similar reductions in the MDA level after exhaustive swimming, while it did not achieve statistical significance.

### Effects of EC and ECC on the activities of antioxidant enzymes

**Fig. 5:**
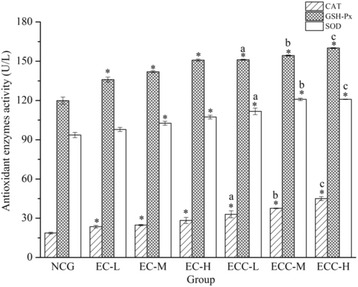
Effects of EC and ECC on the activities of antioxidant enzymes (CAT, SOD and GSH-Px) in mice after exhaustive swimming. Values are expressed as the mean ± SEM. The error bars represent one standard deviation

Except for SOD level in EC-L group, for the EC- and ECC-supplemented groups, CAT, GSH-Px and SOD activities were significantly (*P < 0.05*) higher than those in the NCG (Fig. 5). The mice in the ECC-supplemented groups showed better improvement in CAT, GSH-Px and SOD levels than mice in the EC-supplemented groups. Compared with the EC-L, EC-M and EC-H groups, the SOD levels of mice in the ECC-supplemented groups were increased by 14.17% (ECC-L), 17.8% (ECC-M) and 12.65% (ECC-H), and CAT activity levels of mice in the ECC-supplemented groups had increases of 40.09% (ECC-L), 51.59% (ECC-M) and 58.83% (ECC-H). Compared with the EC-supplemented groups, the concentration of GSH-Px in the ECC-supplemented groups showed significant increases (*P < 0.05*) of 11.18% (ECC-L), 8.7% (ECC-M) and 6.12% (ECC-H).

## Discussion

The present study was designed to evaluate the effects of EC and ECC on endurance exercise in mice. The major findings are listed below:

The ratio of organ to body by weight was regarded as the organ index, which has been commonly used in toxicity tests. Organ damage could be evaluated in terms of the brain-body index [31]. Additionally, the interactions of boron and calcium were observed in the spleen-body index [32]. In our study, EC and ECC had no obvious effects on body weights or on the organ index among the groups, suggesting that all mice showed normal growth and that the extruded products had no toxic side effects on the mice during the experiment. In addition, the reason that the lower body weights of mice may be due to the fact that training can be helpful for the digestion of mice.

The fatigue tests including the weight-loaded swimming test, rolling-bar test, pole-climbing test and mouse tail suspension test can be performed to assess athletic ability [33, 34]. Changes of biochemical markers caused by loaded exercise were not completely consistent with sports intensity and not all of markers were suitable for evaluating the anti-fatigue effect. Among these tests, the anti-fatigue effect of EC and ECC can be evaluated by the weight-loaded swimming test, combining with fatigue related indicators. This method is standard as well as accurate, and it has been adopted widely by most of the researchers and China Food and Drug Administration [4, 30, 35, 36]. However, rolling-bar test, pole-climbing test and mouse tail suspension test require the technical equipment and it is not easy for experimenters to operate them expertly. Lactate test is also often used to evaluate the anti-fatigue effect of compounds on mice after exhaustive exercise, while the test requires the blood sample in inner canthus of each mouse before and after exercise. The procedure may be cumbersome and time consuming to test the lactate level of mice. However, in the weight-loaded swimming test, the workload could be standardized by adding the known amount of load to the tail of the animal to reduce the swimming time, and the endurance time was then recorded accurately, which would be practical to achieve accurate measurements in time [29]. The average of exhaustive swimming time for 4-week-old C57BL/6 mice is 42.2 min (flow rate is 7 l/min) [37]. In our study, after the weight-loaded swimming test, the remaining mice in each group were exhausted in weight-unloaded swimming within 90 min following the standard of exhaustion, which could lessen the effects of stimulation and interference on mice and accord with the generating mechanisms of fatigue. In addition, the concentrations of relevant indicators such as serum CK, BLA, BUN and glycogen, could be raised in exhaustive exercise and it will help improving the quality of detection [38].

For anti-fatigue experiments, the weight-loaded swimming test has been widely used to provide a more informative evaluation of exercise tolerance in animals [39]. Thus, the improvement in exercise endurance will be strong evidence for the anti-fatigue effect [29]. In our study, the results demonstrated that mice in the EC- and ECC-supplemented groups had prolonged swimming time, suggesting that EC/ECC could resist fatigue and ECC supplementation had the most significantly efficient effect, especially at the dose of 20 g/kg. These evidence collectively suggested that ECC-H group had the greatest (*P < 0.05*) anti-fatigue effect among the groups and *C. militaris* could effectively enhance the stamina of mice and delay the emerging fatigue [40]. Stress response represents the reaction of the body and disturbance of normal physiologic balance, along with physiologic changes including the increase of LDH, glucose, cholesterol levels. Moreover, the glucose level of mice could be increased in response to acute perturbations in the environment and it is helpful to increase exhaustive swimming time [41]. Thus, effects of EC and ECC on the recovery from exhaustion could be related to a resistance to the stress in exhaustive swimming. Most researchers choose Kunming, C57BL/6 and BALB/c mice (6–8 weeks) to establish fatigue model [4, 42, 43]. In our study, all of the mice had been forced to swim for 20 min after each treatment so as to be accustomed to swimming, Thus, the ages of mice would make a little impact on stress response.

Glycogen was used for long-term energy storage and could be consumed quickly to satisfy the emergent need for glucose when fatigue occurred [44]. Increasing glycogen levels in the liver and muscle could enhance endurance during exhaustive exercise [45]. EC and ECC are rich in proteins and carbohydrates, which could contribute to the storage of glycogen [42, 46]. Muscle and liver glycogen level increased with the increasing EC and ECC concentrations, which showed that the anti-fatigue activity of EC and ECC might be related to the improvement in the metabolic control of exercise and to the activation of energy metabolism [41]. There is also evidence showing that increasing the availability of fatty acids is mediated by a deceleration of glycogen depletion [43, 47]. *C. militaris* in ECC may decrease carbohydrate utilization by increasing lipid utilization during exercise, which could delay the rate of glycogen consumption. In addition, during the exhaustive exercise, myocytes will consume continuous anaerobic ATP which can be generated via oxidative phosphorylation in the contractile muscle. Under the above conditions, glycogen will be converted into glucose to generate ATP [48]. The aqueous extracts of *C. militaris* administration could increase ATP levels of mice after exhaustive swimming [19]. In our study, *C. militaris* in ECC may reduce glycogen consumption by increasing the concentration of ATP which would be helpful for mice to increase endurance in exhaustive swimming.

LDH activity indicates the degree of lactate metabolism, and the anti-fatigue activity could be evaluated by measuring LDH levels [49]. LDH has the ability to quench BLA, which is derived from the anaerobic metabolism of glucose during high-strength exercise [45, 50]. The data suggested that extruded products, especially *C. militaris* in ECC, could be beneficial to accelerate the metabolism of BLA by effectively strengthening the activity of LDH, which would effectively delay the generation of fatigue [19]. The CK level plays an important role as an indicator of fatigue [38]. An increase in the CK level may be due to damage of the skeletal muscle cell membrane induced by exhaustive exercise [51]. In our study, the results indicated that a high dose of *C. militaris* in ECC could make great contributions to anti-fatigue activity by accelerating the elimination of lactic acid from cells and by efficiently utilizing energy with potential scavenging of free radicals [37].

BLA is the fermented product of carbohydrates without oxygen, and BLA levels increase during anaerobic exercise [26, 41]. In addition, BLA decreases pH and affects the function of cardiac circulation and skeletal muscle systems [52]. The results showed that mice in the ECC-supplemented groups, especially those mice in the ECC-H group, showed a reduction in muscular lactate levels and an increased relative contribution of aerobic metabolism to ATP production during an exercise session [51].

When the body cannot achieve enough energy by metabolizing sugar and fat after a long period of intensive exercise, protein may contribute to the energy supply, and urea nitrogen also increases at this time, which could decrease the strength of the muscle and eventually cause fatigue [52, 53]. *C. militaris* extracts showed a significant suppressive effect on BUN, creatinine and urine protein levels in diabetic mice [54]. In our study, mice supplemented with ECC-H (20 g/kg) had the lowest BUN concentration, demonstrating that the addition of *C. militaris* to the EC could relieve fatigue by slowing down the metabolism rate of protein effectively.

Some diseases are relevant to oxidative damage caused by lipid peroxidation, which may produce harmful metabolites [55]. MDA is a product of lipid peroxidation, and its elevation indicates oxidative damage to the cell membrane [56]. Mice that were administered *C. militaris* polysaccharides at a high dose (0.2 g/kg body weight) had a strong ability to reduce various oxygen free radicals and prevent oxidative stress [53]. In the present study, the rising BUN levels after swimming in the NCG and EC-supplemented group clearly indicated that ECC possessed an ability to lower or retard MDA formation after exercise.

The primary antioxidant enzymes include SOD, CAT and GSH-Px. SOD catalyzes superoxide radicals to produce H_2_O_2_ and O_2_. CAT and GSH-Px catalyze the breakdown of H_2_O_2_ to form water and O_2_ [45]. These antioxidant defense mechanisms become weaker during chronic fatigue and other diseases [57]. Hence, an increase in these defense mechanisms can help to recover from fatigue. In our study, the data showed that ECC-H had the most significant effects on promoting increases in the activities of these antioxidant enzymes. At present, *C. militaris* polysaccharides have been found to protect tissues against oxidative damage in an experiment with mice [53]. Mice in the ECC-supplemented groups possessed a higher capability to prevent lipid oxidation and to preserve cell function by promoting SOD, CAT and GSH-Px levels, indicating that ECC had a better anti-fatigue effect on mice by improving the function of anti-oxidation [58].

## Conclusions

In the present study, EC and ECC had prominent anti-fatigue properties. Our results showed that mice in the ECC-supplemented groups, especially in the ECC-H group (20 g/kg), had more significant anti-fatigue effects. It suggested that adding *C. militaris* could enhance the anti-fatigue effect of EC. ECC enhanced the metabolic control of exercise and activated energy metabolism by increasing muscle and hepatic glycogen levels. In addition, *C. militaris* in ECC may decrease the depletion of glycogen by increasing the availability of fatty acids and ATP level of mice. ECC also efficiently eliminated BLA and protected cells by increasing LDH levels and decreasing CK levels. ECC contributed to the reduction in protein consumption by decreasing BUN levels. Additionally, ECC improved endogenous cellular antioxidant enzymes in ECC-supplemented mice by decreasing MDA levels and increasing CAT, SOD and GSH-Px activities.

In summary, the current study demonstrated that supplementing mice with EC or ECC could promote the capacity for endurance exercise and accelerate recovery from fatigue. ECC had shown the most dramatic anti-fatigue effect, especially with ECC-H (20 g/kg), suggesting that ECC could potentially be included in functional foods for fighting fatigue.

## Abbreviations

BLA: blood lactic acid
BUN: blood urea nitrogen
*C. militaris*: *Cordyceps militaris*
CAM: Complementary and alternative medicine
CAT: Catalase
CK: Creatine kinase
EC: Extruded products of cereal grains
ECC: EC mixed with *Cordyceps militaris*
ECC-H: High dose of ECC
ECC-L: Low dose of ECC
ECC-M: Middle dose of ECC
EC-H: High dose of EC
EC-L: Low dose of EC
EC-M: Middle dose of EC
GSH-Px: Glutathione peroxidase
LDH: Lactate dehydrogenase
MDA: Malondialdehyde
NCG: Negative control group
SOD: Superoxide dismutase
